# Help-seeking for Intimate Partner Violence and Abuse: Experiences of Serving and Ex-serving UK Military Personnel

**DOI:** 10.1007/s10896-023-00534-6

**Published:** 2023-03-25

**Authors:** Rebecca Lane, Filipa Alves-Costa, Rachael Gribble, Anna Taylor, Louise M. Howard, Nicola T. Fear, Deirdre MacManus

**Affiliations:** 1 Department of Forensic & Neurodevelopmental Sciences, Institute of Psychiatry, Psychology and Neuroscience, King’s College London, 16 De Crespigny Park, London, SE5 8AB UK; 2 Barnet, Enfield & Haringey Mental Health NHS Trust (North London Forensic Service), London, UK; 3 King’s Centre for Military Health Research, King’s College London, Weston Education Centre, 10 Cutcombe Road, London, SE5 9RJ UK; 4 Research Department of Clinical, Educational and Health Psychology, University College London, 1–19 Torrington Place, London, WC1E 7HB UK; 5 Section of Women’s Mental Health, Institute of Psychiatry, Psychology and Neuroscience, King’s College London, Dr Crespigny Park, London, SE5 8AF UK

**Keywords:** Partner violence, IPVA, Military personnel, Veteran, Help-seeking, Social ecological model

## Abstract

**Purpose:**

Intimate Partner Violence and Abuse (IPVA) is as a major health concern globally. The prevalence of IPVA perpetration and victimisation has been found to be higher in military compared to civilian populations. Of concern, help-seeking for other psychosocial difficulties among military communities has been shown to be both limited and challenging, and military personnel could face additional or amplified barriers to help-seeking for IPVA than their civilian counterparts. This study aimed to use qualitative methods to explore the experiences of, and barriers to, help-seeking for IPVA victimisation and perpetration among UK military personnel.

**Methods:**

Thematic analysis was conducted on 40 one-to-one semi-structured interviews with military personnel (29 male, 11 female).

**Results:**

Four superordinate themes were derived, thematically organised according to different levels of the social ecological model: *Military cultural factors; Support service factors; Interpersonal factors*; and *Individual factors.* At a military cultural level, participants described difficulties in help-seeking for IPVA resulting from widespread stigma and hypermasculine attitudes in military communities, minimisation of violence, perceived pressure from chain of command, and fear of consequences of reporting. At a support-service level, participants’ negative views or experiences and lack of awareness of services were also significant in deterring help-seeking. At an interpersonal level, participants recounted how relationships with military colleagues, their partner and their family could be both instrumental or a hindrance to help-seeking for IPVA. At an individual level, lack of insight into IPVA and different forms of abuse were suggested through minimisation of violence and described to contribute to delay in help-seeking. Shame, compounded by multi-layered stigma present at each social ecological model level, was a key reason for delaying or avoiding help-seeking.

**Conclusions:**

The findings indicate the added challenges in help-seeking for IPVA experienced by military personnel and highlight a need for a whole systems approach to improve the provision of support for IPVA in the military serving and ex-serving community to instil meaningful change.

## Introduction

The Covid-19 pandemic has put a spotlight on the high prevalence of Intimate Partner Violence and Abuse (IPVA) in the UK (Campbell, 2020), defined as “*any behaviour within an intimate relationship that causes physical, psychological or sexual harm to those in the relationship”* (World Health Organisation, 2012, p1). Evidence strongly suggests that both IPVA perpetration and victimisation are more prevalent and more severe among military compared to civilian populations (Kwan et al., 2020; MacManus et al., 2022; Rentz et al., 2006; Sparrow et al., 2018). The 12-month prevalences of IPVA experience and perpetration in the UK military (12.80% (95% CI 11.72%-13.96%) and 9.40% (8.45-10.45%), respectively) have been found to be higher than in a comparison civilian cohort (adjusted odds (95% CI): 2.94 (2.15–4.01) and 3.41 (1.79–6.50), respectively; MacManus et al., 2022). This is of particular concern given the widespread, multi-generational impact IPVA has on individuals, families, and services and society more broadly (Campbell, 2002; Johnson, Leone, & Xu, 2014; Jouriles & McDonald, 2015; Oliver, Alexander, Roe, & Wlasny, 2019). Research into the impact of IPVA in military populations is scarce. However, recent UK research has highlighted the impact that IPVA experience can have on victim-survivors who are serving in military or civilian partners of those serving, including not only physical harm but also emotional and psychological damage which can contribute to mental disorders such as depression, anxiety, and PTSD (Alves-Costa et al., 2021; Lane et al., 2022a). This research also highlighted the negative impact on children and families in the households in which IPVA occurs as well as wider impacts on the military workforce in terms of fitness to work and operational readiness. No research to date has explored help-seeking for IPVA among military personnel to understand their experiences and highlight service provision, care pathways and barriers to service access.

## Help-seeking and the Social Ecological Model

Applying a social ecological model to help-seeking offers a framework to holistically explore the multitude of factors that affect health behaviour (Golden & Earp, 2012). It has been widely used in health behaviour research (e.g., Alhomaizi et al., 2018; Crosby, Hsu, Jones, & Rice, 2018), and is useful for guiding complex interventions given its broad view of the multi-level factors which influence health behaviour.

Ecological theory posits that beliefs and behaviours must be considered and understood through the lens of the various contexts in which individuals are situated, rather than simply being the product of an individual’s personal attributes (Bronfenbrenner, 1977). The social ecological model, borne from ecological theory (Bronfenbrenner, 1977), outlines how multiple systems and factors (individual, interpersonal, community/organisational, societal/cultural) interact to influence an individual’s views, behaviours and experiences of the world (McLeroy et al., 1988). The societal or cultural level includes factors relating to the greater social, cultural, economic or policy structure within which an individual is found. The societal or cultural context of the social ecological model applied in this research is the UK military. The community or service level encompasses factors relating to structural, organisational and service characteristics and ways of working, as viewed by service users or service providers. In this research, the service level encompasses factors related to both military and civilian support services for IPVA given the focus on help-seeking. Interpersonal factors include relationships with formal and informal social networks. The individual level involves exploring characteristics and internal processes of the individual (e.g., emotional experiences, skills, beliefs, behaviours). Different levels of social ecological model interact in a bidirectional manner; levels are both governed by the wider social context (i.e., top-down influence) and shape the wider social context (i.e., bottom-up influence).

## Help-seeking for IPVA in the General Population

Barriers and facilitators to accessing support for IPVA have been well-documented in civilian samples and highlight the complexities of the help-seeking experience. In keeping with the structure of the social ecological model, barriers have been identified at a societal or cultural level, particularly relating to stigma (Overstreet & Quinn, 2013). Barriers have also been acknowledged at service or institutional level from both a service-user and a service-provider perspective, including lack of awareness of and negative attitudes towards services (Fugate et al., 2005; Huntley et al., 2019), and lack of staff training and confidence to identify and manage IPVA (Ramachandran et al., 2013; Rose et al., 2011; Sprague et al., 2012). At an individual or interpersonal level, numerous barriers including self-blame and fear of repercussions of reporting have been identified (Feder et al., 2006; Patzel, 2001; Rose et al., 2011). As proposed by ecological theory, these barriers do not occur in isolation but rather interact, for instance in the case of cultural, internalised and anticipated stigma (Overstreet & Quinn, 2013), highlighting the significance of cultural context and social norms.

## Help-seeking Among Military Personnel

Much of the help-seeking literature among military personnel has explored help-seeking for mental health difficulties, finding significant barriers to accessing support related to stigmatising beliefs (Cornish et al., 2014; Greene-Shortridge, Britt, & Castro, 2007; Hom, Stanley, Schneider, & Joiner Jr, 2017; Silvestrini & Chen, 2022), mistrust of services and the military (Silvestrini & Chen, 2022) and fears of appearing weak or less able (Cornish et al., 2014; Sharp et al., 2015). Studies have found that military personnel tend to under-report their mental health symptoms and are more likely to prematurely drop out of services than civilian counterparts, exhibiting service-avoidant behaviours (Greene-Shortridge et al., 2007; Hom et al., 2017; Weiss & Coll, 2011). Some research has also focused on military personnel experiences of help-seeking following military sexual trauma (Holland et al., 2016; Holliday & Monteith, 2019; Jackson-Price, 2021; Monteith et al., 2021). Barriers and challenges to reporting and accessing support include how victim-survivors may be perceived by others (Holland et al., 2016), logistical barriers such as time (Holland et al., 2016), poor service experience or minimisation of victimisation experience (Jackson-Price, 2021) or perceptions of institutional betrayal (Holliday & Monteith, 2019; Monteith et al., 2021). With known underreporting of difficulties among personnel and delays and challenges to help-seeking, more must be understood of barriers to reporting and help-seeking for IPVA in order to ensure care provision is appropriate and accessible.

## Help-seeking for IPVA Among Military Communities

Despite the high prevalence of IPVA perpetration and/or victimisation among military personnel (MacManus et al., 2022), the limited research exploring help-seeking and IPVA among military communities has focused on the civilian victim-survivor experience (e.g., Alves-Costa et al., 2022; Gray, 2015; Williamson, 2012; Williamson & Matolcsi, 2019) or health professional perspectives (Sparrow et al., 2020). Evidence emerging from this body of literature strongly suggests that barriers to help-seeking for IPVA may be shared with general population samples whilst also being complicated by or amplified as a result of the military context (Alves-Costa et al., 2022; Sparrow et al., 2020). For example, civilian victim-survivors of abusive relationships with military personnel have highlighted that in addition to fear of their (ex)partner or self-blame narratives, the perceived normalisation and minimisation of violence in military environments and a perceived lack of confidentiality at the intersection between professional and personal spheres were key factors influencing their motivation and ability to seek help (Alves-Costa et al., 2021; Gray, 2015, 2016; Williamson, 2012; Williamson & Matolcsi, 2019). Military experiences, including frequent relocations, transitions and deployments can isolate military couples from support networks (Alves-Costa et al., 2021, 2022; Lane et al., 2023), and have been shown to obstruct avenues to help-seeking among civilian partners in abusive relationships with military personnel (Alves-Costa et al., 2022). In addition, public perceptions of the military, perceived military protection of personnel and the lack of coordination between civilian and military judicial services were additional barriers highlighted by civilian victim-survivors (Alves-Costa et al., 2022), highlighting the social and cultural challenges of help-seeking for IPVA in military communities.

Barriers to help-seeking for IPVA among military personnel (who may be perpetrators and/or victim-survivors) may be uniquely complex. From a health and welfare professionals’ perspective, it is perceived that military cultural factors, such as the hypermasculine environment of the military, ideals of strength and stoicism, are associated with significant under-reporting of IPVA among personnel (Sparrow et al., 2020). More is required from a military personnel perspective to understand the likely multiplicity of barriers to help-seeking for IPVA arising and interacting at cultural, service, interpersonal and individual levels. A particular gap in research is the experiences of personnel who have experienced victimisation, especially the experiences of male victim-survivors, which few studies have explored (Sparrow et al., 2018).

## The Present Study

To the best of the authors’ knowledge, no research has explored attitudes to and experiences of help-seeking for IPVA (perpetration, victimisation, or both) from the perspectives of military personnel. An in-depth understanding of military personnel experiences of help-seeking for IPVA is needed to inform the development of care provision and support and would significantly benefit upcoming reviews of the MOD Domestic Abuse Strategy (2018). This study aims to address this gap by qualitatively exploring military personnel’s experiences of help-seeking for relationship difficulties and IPVA perpetration and victimisation and identifying barriers to accessing appropriate support, in particular military-specific barriers.

## Methods

### Study Design

This study is a qualitative study using semi-structured interviews with UK serving and ex-serving military personnel. It forms part of a wider mixed-methods research programme aiming to better understand IPVA within the UK military community (Alves-Costa et al., 2021, 2022; Lane et al., 2022a, 2022b, 2023; MacManus et al., 2022). A social ecological model is used to understand help-seeking for IPVA in the military community in the UK. As such, the different levels of the social ecological model are embedded within a military societal context, with military culture representing the highest level of the model. As this research is focused on help-seeking for IPVA, the community or organisational level of the social ecological model encompasses views, experiences and characteristics of, as well as pathways to, services/organisations offering support.

## Definitions

In this paper, we used the term *bidirectional abuse* to describe experiences of participants who report relationships with mutual violence, whereby both partners engaged in abusive behaviour toward the other. This may be symmetrical or asymmetrical in terms of severity, but both partners are considered to instigate abusive behaviours and the behaviours are not suggestive of perpetration or victimisation only with retaliation (reaction to relationship violence or abuse with the intent of fighting back) or resistance (response to relationship violence or abuse as a last resort, with the intent of defending oneself from injury). We used the term *unidirectional abuse* for cases where participants report perpetrating IPVA towards their partner or being a victim-survivor with no reports of bidirectional violence. Unidirectional abuse may occur with or without instances of retaliation or resistance.

## Recruitment

Participants were recruited from a sub-sample of respondents to phase 3 of the King’s Centre for Military Health (KCMHR) Health and Well-being Cohort Study, which collected information on the health and well-being of personnel deployed to Iraq and Afghanistan between October 2014 and December 2016 (Stevelink et al., 2018). The recruitment pool for Phase 3 included those who participated in Phase 1 or 2 and consented to be followed up (n = 12,280), in addition to a replenishment sample of trained regular and reserve personnel who joined the military on or after 1st August 2009 and were in service on 31st March 2013 (n = 8581). For full details of the sampling and response rates for Phase 3 data collection and recruitment, please see Stevelink et al. (2018).

Within the phase 3 KCMHR self-administered survey, participants reported on experiences of victimisation and/or perpetration of IPVA in the previous 12 months, including psychological, emotional, physical and sexual abuse (MacManus et al., 2022). A total of 8,093 participants completed the Phase 3 survey, of which 5,557 reported a relationship in the previous 12 months. Of the 5,557 reporting a relationship, 469 reported any form of IPVA perpetration and 689 reported any form of IPVA victimisation within the previous 12 months (MacManus et al., 2022). Of those who endorsed either IPVA perpetration or IPVA victimisation, or both, 266 (188 men and 78 women) consented to be followed up and were invited to take part in the current study via email and postal letter. Prior to study involvement, participants received study information and provided written consent.

## Materials

The topic guide captured participant demographic, relationship and military characteristics and explored participant experiences and attitudes regarding help-seeking for IPVA, as well as participant suggestions on what they found/would have found helpful. A mixture of closed and open questions were asked to elicit participants’ experiences of help-seeking; perceived facilitators and barriers they experienced to access support for IPVA; military specific factors which affected their help-seeking journey; and usefulness of care provision from civilian and military services and recommendations for policy and practice. Example questions include: *have you ever sought help or talked to someone about the difficulties that you had in your relationship?; were there any reasons that might have stopped you from seeking help?; if you were to have sought support, what support do you think would have been helpful?; in general, how would you describe help-seeking among military personnel?.* Interviews lasting between one to two hours were conducted via telephone between January to August 2018. All interviews were audiotaped/recorded and transcribed smart verbatim. Participants were offered £25 as compensation for their time.

## Analysis

The data analysis was conducted using thematic analysis (Braun & Clarke, 2019, 2020). Data management was supported by QSR NVivo12 software (QSR International, 2018). Following the transcription and *familiarisation* with the data, and during the *indexing* or *coding* phase, an initial *thematic framework* was constructed through open coding and priori themes from the literature, akin to that used in a Framework method (Ritchie et al., 2013). *Charting* the data allowed for understanding the data at a high level and creating a more manageable dataset to take forward into the next stages of analysis and interpretation. A priori themes from the literature and interview schedule were introduced into the coding schema, such as ‘awareness of services’, ‘stigma’ or ‘lack of confidentiality’ but were retained in the dataset only where supported by participant narratives. Additional codes emerging within the data were integrated into the framework and the framework was continually refined by moving between data and themes. Finally, during *mapping*, categories and dimensions were identified. These were organised and developed into themes and subthemes by iteratively revisiting data and codes and through discussions among the research team. Two co-authors (FAC and AT) conducted the initial *coding* and *charting* of the data, which was subsequently reviewed by a third co-author (RL) to finalise the *charting* stage and complete the *mapping* stage. Comparisons across sub-groups (e.g., gender, IPVA status, branch of Service, serving status, rank) were considered and reported where relevant.

## Efforts to Enhance Rigor

The research benefitted from patient and public involvement (PPI). A PPI group was developed involving consultation with professionals (military research, IPVA research and services, mental health research and services, members of the Armed Forces) and civilians with personal experience of abuse by their military (ex)partners. Feedback from this group helped inform the interview protocol and develop the framework. PPI events were also organised to gain feedback on the findings, which allowed the results to be refined, verified and validated.

The research team have engaged in reflexive processes throughout the study design, data collection and analysis. The research team’s interactions with participants and interpretations of the data might have been influenced by their own identities, experiences and prior assumptions. Interviews were conducted by two authors (AT and FAC), who then went on to complete the first stage of the analysis (initial coding and charting of codes). The analysis was completed by an author independent to data collection (RL; charting and mapping), which allowed for triangulation and convergence of researcher interpretations, and validation of themes and subthemes within the framework. Throughout the analysis process, themes and subthemes were reviewed through progressive iterations and discussions within the research team and PPI groups. The varied experiences of the researchers and those consulted with were beneficial to the narrative and understanding of the data.

## Ethics

Ethical Committee approval was obtained from Ministry of Defence Research Ethics Committee (823/MODREC/17). Due to the sensitive nature of the interview, a risk management plan was developed. All participants were offered the opportunity to discuss any concerns following their interview with an independent clinician and were signposted to support services.

## Findings

A total of 40 participants, 29 men and 11 women, aged between 24 and 65 years took part in the study, see Table 1. The majority of participants described themselves as White British; those who did not, identified as Asian-Bangladeshi, African, Black Caribbean and Chinese-Asian. Many participants were in the Army, ex-serving personnel, of Non-Commissioned Officer (NCO) rank, Regular personnel and had previously deployed. Most participants reported heterosexual relationships, with two participants reporting same-sex relationships.

**Table 1:** Participant demographic, relationship and military characteristics

	*n* (%)
**Age** (years)	
*< 35*	7 (17.5)
*35–49*	22 (55.0)
*50+*	11 (27.5)
**Ethnicity**	
*Minority ethnic group*	4 (10.0)
*White*	36 (90.0)
IPVA reported*	
*Reporting IPVA victimisation*	27 (67.5)
*Reporting IPVA perpetration*	17 (42.5)
*Reporting bidirectional IPVA*	29 (72.5)
**Dual military relationship reported**	
*Yes*	16 (40.0)
*No*	24 (60.0)
**Branch**	
*Royal Navy/Royal Marines*	7 (17.5)
*Royal Air Force (RAF)*	11 (27.5)
*Army*	22 (55.0)
**Serving status**	
*Ex-serving (veteran)*	31 (77.5)
*Serving*	9 (22.5)
**Engagement status**	
*Regular*	33 (82.5)
*Reservist*	7 (17.5)
**Rank** (at time of interview or leaving Service)	
*Officer*	7 (17.5)
*NCO*	30 (75.0)
*Other rank*	3 (7.5)
**Length of service** (years)	
*5 to 14*	19 (47.5)
*15 to 24*	11 (27.5)
*25+*	10 (25.0)
**Deployment experience** **	
*Deployed*	35 (87.5)
*Not deployed*	5 (12.5)

* Some participants reported different IPVA patterns across different relationships. As such, these are not mutually exclusive

**Deployment experience does not include detail on whether military personnel held combat roles on deployment, although participant narratives would suggest this was common

All participants recruited had reported IPVA experiences, either perpetration, victimisation or both, within the last 12 months in the KCMHR Military Health and Wellbeing questionnaire study (Stevelink et al., 2018). In the interviews, participants described lifetime IPVA experiences, sharing a range of patterns, severity and types of IPVA across different relationships. As such, patterns of abuse are not reported as mutually exclusive and some military personnel have reported unidirectional victimisation and unidirectional perpetration of IPVA in the context of different relationships, as well as bidirectional IPVA (see Lane et al. (2022a) for detailed analysis of experiences and impact of IPVA in this cohort). Where possible, quotes are accompanied by labels depicting the IPVA patterns within the relationship relevant to the context of the quote.

Sixteen participants (11 males and 5 females) reported seeking help from military (n = 10) or civilian (n = 10) services for their relationship difficulties, with four reporting seeking help from both. Of note, only one participant reported the police being involved and no participants reported pressing charges/being charged for abusive behaviours within their relationships. The majority of participants (n = 24) reported no help-seeking for their relationship difficulties from either civilian or military services, with some expressing an ongoing reluctance to seek help in the future should relationship difficulties arise or persist. No group differences in sociodemographic characteristics or IPVA experiences were observed among those who sought help for their relationships and those who did not. Some participants reporting IPVA perpetration received support for their mental health or anger management difficulties. None were referred to perpetrator programmes.

**Fig. 1:**
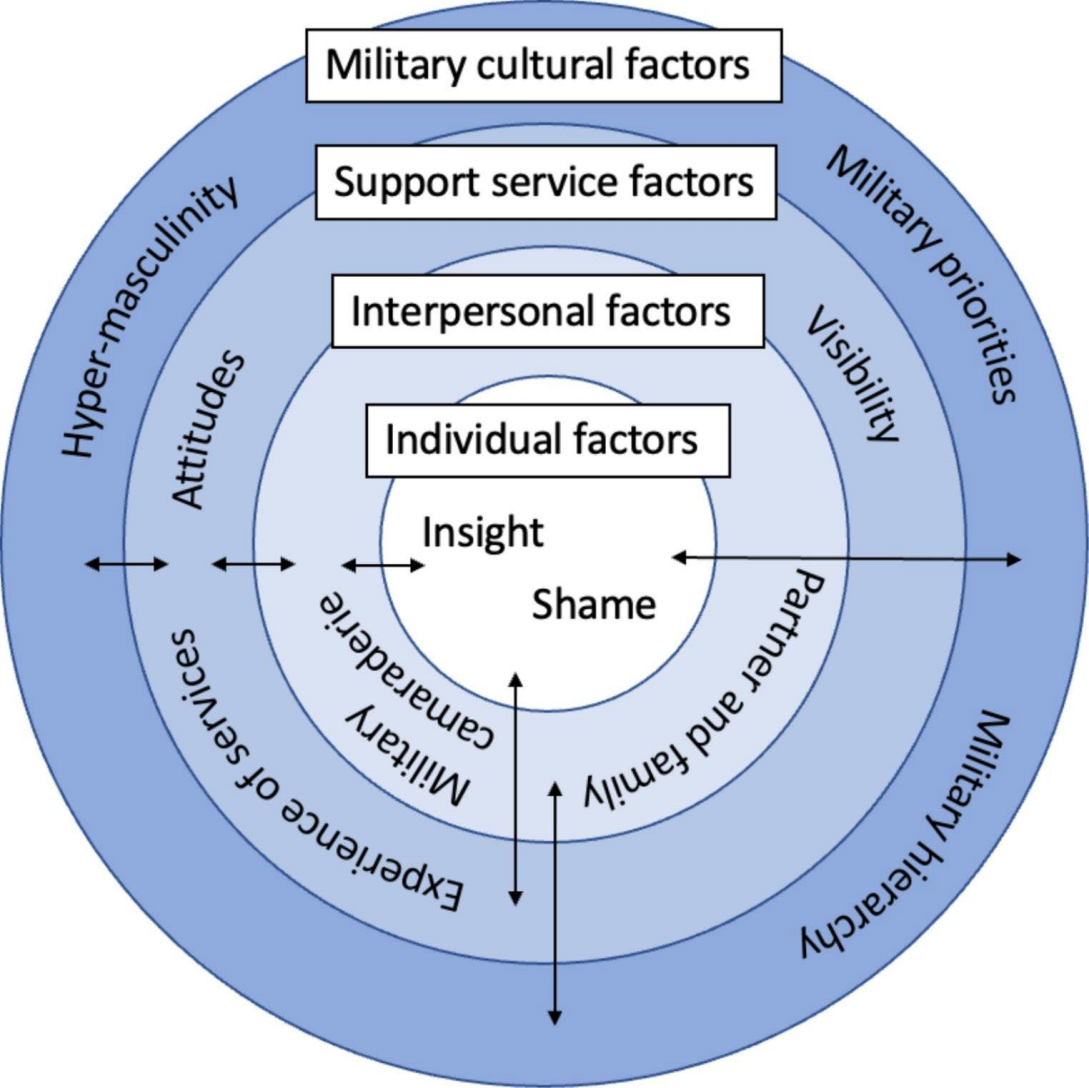
Interrelatedness of themes and subthemes illustrated within the social ecological model applied to this study

The findings describe participants experiences of help-seeking and how different factors were perceived to affect their help-seeking behaviours and experiences. These were thematically organised according to the four levels of the social ecological model applied to our research, which are situated within the ecosystem of the UK military society and focussed on drivers and barriers to and experiences of help-seeking from support services, as well as informal sources of support. The four overarching themes include: *Military cultural factors; Support service factors; Interpersonal factors*; and *Individual factors*, see Fig. 1. Many reported help-seeking experiences in relation to mental health which they perceived to be impacting or a consequence of their relationship difficulties, discussing these interchangeably with experiences of help-seeking specifically for abusive behaviours within their relationships. As a result, the following results describe drivers, barriers to and experiences of help-seeking more broadly among military personnel, with a focus on help-seeking for relationships and IPVA.

### Theme 1: Military Cultural Factors

Theme 1 speaks to factors at the highest level of our social ecological model, the military cultural level, which were perceived to affect help-seeking behaviours.

#### Hyper-masculinity

Despite acknowledging the availability of services for military personnel, most participants described barriers to help-seeking for relationships relating to cultural stigma surrounding hyper-masculinity and a desire to not appear weak (also see Theme 4 - *Shame*). This was perceived by participants to be reinforced through military training, heightened among certain military units, and perpetuated through interactions with colleagues and chain of command.

*The forces is very macho, still a very male-dominated environment. When I [sought help] in Afghanistan, for me to have to go to my commanding officer and say, ‘Look, my marriage is in a mess. I need to get home,’ that took me weeks to get to that point because that is the mindset. […] If you are more worried about your home life than you are your operational tour, you lose effectiveness and, for us, that means losing credibility. If you lose credibility, you might as well jump off a bridge. [P29; Male; Army; IPVA perpetration with victim retaliation reported]*

Hyper-masculinity among military personnel was described to discourage discussions of emotions and denote a perceived resilience and a responsibility to cope independently. Some perceived help-seeking to be socially acceptable only if experiencing acute or severe difficulties. Although hyper-masculinity was identified as a barrier for many participants, this was especially prominent among Army personnel, participants who were ex-serving, and those who had previously deployed.

*People in the military generally ask less for help. […] you have always taught that you have got to cope with the most stressful situation in your training. […] It feels like, right now, the only way of getting that support [for relationships] is if it is something extremely significant going on. [P11; Female; Navy; Dual military relationship; IPVA victimisation with retaliation reported; help-seeking for relationships from military services reported]*

#### Military Priorities

Many participants shared a view that military priorities were put before the needs of personnel and families, which impacted their ability and motivation to seek help and could result in a perceived lack of support. This was described to vary according to chain of command, role and unit, with participants reporting more challenges for reservists, those of higher rank and those in small units (also see Theme 3 – *Military camaraderie*). Military priorities were perceived by participants to dictate the military’s ability and willingness to help, with some sharing that the military intervened only when military priorities, such as operational effectiveness or maintenance of the family unit, were at risk. Among those who did not seek help from the military, de-prioritisation of personal difficulties and limited opportunities or time to seek help were identified to be significant.

*With experience, I have come to realise that, despite all the good things they say, the military really has very little interest in the welfare of the individual or families. It is very much they will prioritise the work perspective, and occasionally comes up with nice words, but, in practice, the priority is almost always given to what the military wants to achieve. [P6; Male; RAF; bidirectional abuse reported]*

*There is no time to talk to [military personnel] for a start, because they are rushing to exercise, they are rushing to even training, and he [chain of command] is dealing with a million things. There is no time to really talk about anything else [relationship difficulties]. [P3; Male; Army; IPVA victimisation reported; help-seeking for relationships from civilian services reported]*

#### Military Hierarchy

Participants identified the chain of command as gatekeepers to accessing support within the military community. A few described how their immediate managers were instrumental in signposting them to appropriate support and granting time off work to seek help, noting that helpfulness depended largely on the individual manager. Others described that their impersonal relationships with their managers were not conducive to asking for help, particularly for reserve personnel, and reported a lack of confidence that help would be provided. Some participants described additional challenges to help-seeking due to their rank as Officers, whereby an increased sense of responsibility and a focus on the needs of the military and of others were barriers to their own efforts to seek help.

*My immediate boss has been the most supportive person I could have ever asked for. […] He has been here 20 plus years, so he completely understands my side, and very useful to support me. And my peers were. Because there is quite a large hierarchy in the navy, when it went above my immediate boss, no one seemed to care at all. [P1; Male; Navy; bidirectional abuse reported; help-seeking for relationships from military services reported]*

*I only ever had contact with welfare services if I had a squadron commander or flight commander looking after my troops. It never really occurred to me that they were there for me as well. [P6; Male; RAF; bidirectional abuse reported]*

### Theme 2: Support Service Factors

Theme 2 describes factors influencing help-seeking which were associated with the support services or occurring at the community or organisational level of the social ecological model.

#### Visibility of Support Available

Some participants reported being unaware of where, how and for what they could access help for their relationships. A perception that the military is not forthcoming in advertising help available or offering solutions to difficulties was especially pronounced among ex-serving and reservist personnel, and in relation to support for spouses and families.

*I don’t know if it is ignorance on my part that there is anything there [for my partner], but it is certainly not publicised. […] I know, again, the regulars have counsellors or whatever that they can go and talk to, but I certainly wasn’t aware that there was anything available to me [as a reservist]. [P23; Male; Army; IPVA victimisation with retaliation reported]*

#### Attitudes Towards Seeking Formal Support

Negative perceptions of services, including prior negative experiences with services or a lack of confidence in the value of support, were described by a few participants as primary reasons for not seeking help. This appeared more prominent in narratives describing support for relationship in comparison to mental health difficulties.

*Sometimes we recover from an argument fairly quickly. It has definitely been spoken about – counselling and things – in the past. I think the worry is, though, for both of us, if we are already alright and we go to counselling, it could possibly cause more problems. So neither of us bought into it fully at the time [P1; Male; Navy; bidirectional abuse reported; help-seeking for relationships from military services reported]*

Many feared lack of confidentiality within the military community and anticipated stigma and negative response from others should it become known they experienced relationship difficulties for which they sought help. The interlinked nature of military and health records were significant barriers to seeking support, in particular the perceived impact help-seeking could have on participants’ careers. This was reported to impact help-seeking for both relationship problems and/or related mental ill-health and was a particularly prominent theme among older participants. In response, many advocated for a confidential helpline or the opportunity to speak with practitioners separate from the military, but with military knowledge.

*If you had something, you would go and talk to one of your mates or chew the cud with them over a beer […] but taking that a bit further, it always seemed to be the case of you couldn’t do confidentially; you would have to do it through a chain of command. […] So, if you went to your manager and you said, ‘I’ve got relationship difficulties,’ or whatever, it is like a little tick in the box about delaying your deployment. […] So, rather than saying anything at all about any mental illness, relationship difficulties, anything at all, it is just stay schtum. [P25; Male; RAF; IPVA victimisation, perpetration and bidirectional abuse reported]*

*I couldn’t say that they would break confidentiality or anything else, but anything embedded with you, you always have to be careful – ‘Why is Private X going down to the SSAFA sister 10 times this week?’ and people will ask questions. All it needs is the wrong word at the right time, and people will still see it as finishing their career. [P2; Male; RAF; IPVA victimisation and bidirectional abuse reported]*

#### Experience of Services

Services utilised by participants included military health and welfare services, civilian health services and civilian or military mental health or IPVA-related charities, such as Combat Stress and RELATE. Participants who had accessed services for their relationship difficulties, IPVA and related mental health difficulties reported both positive and negative experiences, often holding polarised views. Only a minority received support to leave abusive relationships. Feeling listened to and having a third-party opinion were described by some to be especially helpful when dealing with relationship problems, with a few calling for the implementation and advertisement of anonymous groups for personnel to share common experiences. This appeared a more prominent theme among those who had previously deployed and among ex-serving personnel, who highlighted the importance of feeling understood. Furthermore, among those who received support for their mental health difficulties related to their relationship conflict, some observed improved emotion regulation and communication with their partners, positively impacting their conflict resolution strategies.

*[SSAFA] told me to bin them, really, to leave, because she was the abusive cheating one. I was blaming myself. […] I had support groups. I had everything like that. They gave me numbers, they gave me emails, they gave me websites. Absolutely amazing. [P8; Male; RAF; IPVA victimisation with retaliation reported; help-seeking for relationships from military services reported]*

*At some points, up until when I actually went and got some help [for PTSD], [our relationship] was very, very bad. But now, because I have got some help, everything is really good, because we both know now what we have got to do to cope with it, and I know I have got to talk to my partner. [P24; Male; Army; IPVA perpetration, IPVA perpetration with victim retaliation reported]*

However, many reported negative experiences. Among those who sought help from civilian services, some reported that the support they received was not specialised, echoing an assumption described by many that civilian services could offer little support due to a lack of first-hand understanding of military experiences. Some also expressed that long waitlists were not conducive to receiving timely support and short appointments or consultations discouraged disclosure. Additional barriers relating to security concerns were highlighted by some due to the classified nature of their role in the military, limiting who they could seek help from.

*[Both] counsellors, they are good at what they do relationship-wise, but I think they lack the understanding of the stresses that military life imposes on couples. [P34; Female; Army; bidirectional abuse reported; help-seeking for relationships from civilian and military services reported]*

*So we agreed that I would get the counselling to deal with the military side of it and, if that didn’t sort it, we would get the couples counselling, because then it would be more relationship-based. Obviously, with being part of the organisation I was, there are certain things I can and cannot discuss because people have to have security clearances for it. [P24; Male; Army; IPVA perpetration with victim retaliation reported]*

Some participants reported a preference to seek help from military services, whilst others shared that welfare officers are not trained as counsellors. Some participants described negative experiences of help-seeking from military services, including a slow response, lack of enquiry about abuse and lack of follow up or signposting after relationship or related mental health difficulties were disclosed. These difficulties were reported to be compounded by frequent military-related relocations. A few participants described positive experiences of receiving help from the military but noted that support was largely short-term and practical, for instance relieving some relationship stressors by providing childcare or housing, but not addressing other more causally related factors such as working patterns and stress, separation or alcohol or mental health difficulties. Furthermore, despite recognition that participant accounts were retrospective and may not reflect change within the military in the last decade, some participants perceived change to be slow and reported that an institutional shift to make help-seeking more acceptable is still required.

*I did receive good support [from the military] to help things with [partner], but the root cause that is coming from work they haven’t really assisted with that at all. […] I rang up the welfare system […] and they turn to you and say, ‘Well, it could be five working days until we get back to you,’ […] I just said the relationship is breaking down, it is causing me to have problems at work, and there was nothing majorly urgent. […] They didn’t ask about [physical aggression], and I didn’t [tell them]. [P1; Male; Navy; bidirectional abuse reported; help-seeking for relationships from military services reported]*

*The first time [we sought help for our relationship] it was myself who spoke to my welfare officer, but they said they didn’t fund that sort of thing, which was a lie at the time. So we paid for our own. They gave me signposting details. […] It was just almost like a fleeting discussion. […] I was posted shortly after that so there was no expectation to sort anything out for me. [P34; Female; Army; bidirectional abuse reported; help-seeking for relationships from civilian and military services reported]*

*I have not seen, in my 20-odd years, great progression through it, the stigmatising of asking for help. I think that is a very difficult thing to overcome. What will happen is it will change with the younger ones coming in, because, if you come in now and you get used to a culture of, yes, you can [ask for help] and it will be alright, as they progress through the ranks, they will make sure that the people below them know that. Whereas I am probably a product of the 80s where you will still see it. When I turned up, it was very much along the lines of, ‘You, get up there or you are going to get a thumping,’ […] so you did. You didn’t question it. You got on with it. [P2; Male; RAF; IPVA victimisation and bidirectional abuse reported]*

### Theme 3: Interpersonal Factors

Theme 3 describes factors perceived to be associated with help-seeking which relate to interpersonal relationships.

#### Military Camaraderie

Although many reported not sharing their relationship, IPVA or mental health difficulties with their friends and colleagues due to shame (i.e., internal and anticipated stigma), some participants expressed the value of informal support among personnel as an alternative to formal help-seeking, borne from close knit relationships and shared experiences and understanding. This was especially reported in the case of mental health difficulties. Many reported informal support from military friends and colleagues in the context of using alcohol. Moreover, a minority noted camaraderie within the military and having challenges in common can normalise some of the relationship difficulties and/or related mental health problems experienced, such as self-medicating with alcohol despite its contribution to IPVA and relationship difficulties, as illustrated below.

*There is a lot of forgiveness in the military and there are a lot of people who are very like you [who use alcohol to cope]. […] So there is always someone there who will agree with you, feel sorry for you, justify what you are doing even though you might think it is wrong or you might know it is wrong but don’t want to admit it. Whereas, when you are out of the military, […] you don’t have that, and it makes you take much better stock of what you are doing and who it is hurting. [P37; Male; Navy; bidirectional abuse reported; help-seeking for relationships from civilian services reported]*

Furthermore, the sense of camaraderie tended to be limited to close colleagues and understanding welfare officers or managers and was less prominent for reservist and ex-serving personnel. Some participants reported a perceived pressure to not let their peers down and were reluctant to access military support services for fear of them finding out, especially if working in small teams, living on base or having embedded welfare and mental health services.

*I think [help-seeking] is frowned upon, because a lot of people who end up getting the help end up getting knocked off the boat. That leaves those on the boat in limbo. […] It is a nightmare. If there are four of us doing a job and we drop down to three, you have then got to work two weekends in a row to get the following off. […] [the person who sought help] are hated. […] No one really wants anything to do with anyone through that route. [P22; Male; Navy; bidirectional abuse reported; help-seeking for relationships from military services reported]*

#### Partner and Family

A few participants reported their partner being instrumental in helping them access support, particularly for their mental health difficulties, in some cases accepting support for their partner’s sake rather than their own. However, others shared that a lack of willingness in their partner to engage in relationship counselling was a barrier to accessing support, a more prominent theme among female participants and among those in dual military relationships.

*Me personally, I was looking at the idea of relationship counselling and things through Relate or other organisations. I wanted to make that work. I wanted to discuss those problems together, but he was having none of it. [P39; Female; RAF; Dual military relationship; IPVA victimisation with retaliation reported]*

Among those who disclosed more severe experiences of IPVA victimisation, participants shared barriers to help-seeking or leaving the relationship, such as fear of the repercussions of reporting, manipulation/coercion and threats from their partner, persistent love and hope for the relationship, and maintenance of the family unit. Drivers to leaving relationships were reported by some participants to result from heightened experiences of abuse, often physical.

*I was already not seeing my son from my first marriage, so I felt that I owed it to my daughters. I was trying to wait until my youngest was 18 [before leaving]. […] I had left previously for two years […] but my eldest daughter had gone from a straight A student to Cs and Ds. So I moved back, really, to establish her education again. […] I had my sights set on the fact that I would leave there as soon as I could, when the children were able to sustain themselves. [P40; Male; RAF; IPVA victimisation reported]*

*I didn’t stay with him much longer after [sexual assault], to be honest. […] There was always the threat there of [physical aggression] that was what would happen. […] I ended up having to walk for my own safety. […] No [he wasn’t prosecuted]. I was scared by what he might be able to do. [P39; Female; RAF; Dual military relationship; IPVA victimisation reported]*

### Theme 4: Individual Factors

Theme 4 summarises the factors perceived to influence help-seeking for IPVA at the level of the individual.

#### Insight

Despite disclosing experiences consistent with IPVA (perpetration or victimisation or both) within interviews, many participants minimised these behaviours, not perceiving them to be serious or to warrant formal support. IPVA in many narratives was normalised or minimised relative to what they observed among their peers, attributing the difficulties to general stress, or not recognising what can constitute IPVA, especially non-physical forms of abuse. Some noted that time spent away due to the military contributed to their lack of insight. This appeared a more prominent theme among participants who were male and in dual military relationships. Lack of insight was also identified as an important factor in delaying help-seeking for mental health difficulties, which in some cases impacted on relationships significantly.

*Nothing serious. We have threatened to [indecipherable] each other and things like that, but not serious blows. No, there has never been a serious threat on life or anything like that. […] Just threatened to hurt each other now and then. […] nothing ever really comes of it. It is just in the aggression of the moment. [P1; Male; Navy; bidirectional abuse reported; help-seeking for relationships from military services reported]*

*I have certainly come close to [physical aggression] because I am very in your face. […] I can get inside somebody’s space. I know I do that. I don’t know if that is classed as physical. I don’t physically touch. […] I have never struck out. […] Perhaps pushing. Perhaps I have, yes. […] sometimes I have pushed past somebody to get out of the way as well. [P29; Male; Army; IPVA perpetration with victim retaliation reported]*

*We nearly split up over that because it got to the point where I wore her down so much that she didn’t feel like herself anymore. She actually went to a doctor and was on antidepressants for a while, and that was kind of like my kick in the teeth. […] I didn’t have a clue and it was actually when we had an argument it all came out in the wash then. I just took a long hard step back and look at myself, and couldn’t believe what I had done. […] I would say, if I hadn’t had got the counselling […] the relationship would have ended; not in a matter of years, in a matter of weeks. [P24; Male; Army; IPVA perpetration with victim retaliation reported]*

A minority of those reporting experiences of victimisation identified that being young contributed to a lack of insight regarding IPVA, as well as a gradual increase in abusive experiences over time, which fed into self-blame narratives. For some participants reporting either IPVA victimisation and perpetration experiences, instances of heightened aggression and violence at home were triggers to gaining insight into the abusive nature of the behaviours and provided motivation for either help-seeking for mental health and/or relationship difficulties or for leaving the relationship.

*When I was married to my ex-wife, I probably buried every emotion in my body because of what was happening, for the children. […] I blamed myself an awful lot because I allowed it to happen; I allowed her to dictate life to me, but it happened so gradually that I didn’t see it happening. So I felt responsible in many ways, as I should have been stronger, I should have seen it. [P40; Male; RAF; IPVA victimisation reported]*

*What triggered me to go for therapy is when I punched her. I thought to myself, ‘I have a major problem here; I think there is something wrong with me,’ and that is what got me to go into therapy. [P21; Male; Army; IPVA victimisation with retaliation reported]*

#### Shame

Many participants identified that a reluctance to discuss personal issues with others and seek outside support was linked to shame and embarrassment as key reasons for delaying or avoiding help-seeking, largely driven by stigma surrounding IPVA. Some shared a preference to keep matters private, wanting to sort difficulties independently. This was more prominent among participants over 50 years of age.

*[We didn’t ask for help] because it was nobody else’s problem. […] I didn’t want people knowing that I had relationship problems. […] It could have been culture [that prevented me from seeking help]. It could have been ego. It could have machismo. I don’t know. I didn’t want other people meddling in what was my problem. [P26; Male; Army; IPVA victimisation and bidirectional abuse reported]*

Among those reporting more severe experiences of IPVA, especially among male participants, shame in admitting to the perpetration of abusive behaviours or experiences of victimisation was a significant barrier to asking for help or pressing charges.

*I think it was facing somebody and going over my weaknesses as a human, I suppose [was the main reason that I was fearful to speak about relationship problems]. To tell somebody that you would be that angry with somebody that you would be literally three or four centimetres from their face, and a woman, […] to admit that to a third party, face-to-face, just the thought of it now scares the life out of me, quite frankly. [P29; Male; Army; IPVA perpetration with victim retaliation reported]*

*If I am honest, I was totally embarrassed with the situation. […] Stigma. [indecipherable] a soldier and a fairly large chap, it is not right that I have been controlled by this person. [P40; Male; RAF; IPVA victimisation reported]*

## Discussion

This study explored the IPVA help-seeking experiences of military personnel who had reported IPVA (victimisation, perpetration, or both) within a recent relationship. Four main themes were identified representing the different levels of participants’ social context according to the social ecological model: *Military cultural factors; Support service factors; Interpersonal factors*; and *Individual factors.* Barriers to help-seeking and accessing appropriate support for IPVA largely overlap with those identified among civilian populations, including stigma, lack of insight and negative perceptions of support (Huntley et al., 2019; Overstreet & Quinn, 2013), but appear amplified by aspects of military culture and experiences, such as hyper-masculinity and military priorities, in keeping with the narratives of civilian partners of military personnel (Alves-Costa et al., 2022) and those of health and welfare professionals working with serving military personnel or veterans (Sparrow et al., 2020).

As anticipated, stigma and shame were significant barriers to help-seeking for IPVA at all levels of the social ecological model, in particular individual and cultural level stigma as well as anticipated stigma. This echoes IPVA help-seeking research among civilians and help-seeking research among military personnel more broadly (Cornish et al., 2014; Greene-Shortridge et al., 2007; Holland et al., 2016; Holliday & Monteith 2019; Hom et al., 2017; Monteith et al., 2021; Overstreet & Quinn, 2013; Silvestrini & Chen, 2022). Of significance, the stigma associated with IPVA and with help-seeking for relationship difficulties were observed to be perpetuated and magnified by aspects of military culture and the military environment, highlighting in particular the impact of hypermasculine attitudes on the perception and reporting of IPVA (Barstow, 2000; Enloe, 2000; Sparrow et al., 2020; Taylor, Keeling, & Mottershead, 2019) and a need for a military-wide overhaul in how IPVA is recognised and perceived. Indeed, barriers relating to minimisation, denial and poor understanding of IPVA reported by participants, as well as fear of reporting, are likely by-products of substantial stigma and the military cultural context, as found also in the narratives of civilian partners of military personnel (Alves-Costa et al., 2022). Beyond the recognition of abuse, such factors can contribute to delays in or avoidance of help-seeking. Furthermore, many deterrents of help-seeking revolved around fear of stigma arising from the potential impact on unit cohesion, others’ perception of their prioritisation of self over Service, and potential impact on their career, as found in previous research on help-seeking for mental health difficulties among military personnel (Hom et al., 2017).

Participants largely held negative perceptions of asking for and receiving help, chiming with findings on attitudes to help-seeking more broadly among male military personnel (Bass et al., 2016). Pre-conceptions of services were highlighted by participants as barriers to help-seeking, particularly a lack of confidence that services would have an impact or that they would be sufficiently specialised to understand participants’ military experiences. Corroborating previous findings among civilian partners of military personnel (Alves-Costa et al., 2022; Gray, 2015, 2016; Williamson, 2012; Williamson & Matolcsi, 2019), participants identified that having chain of command or military colleagues as gateways to support, or having support located on base, could introduce concerns about confidentiality and entitlement to support (for instance in the case of Officers) and could deter help-seeking. Of note, many participants were not aware of the support available, especially of services for spouses or reservists, groups previously identified as being generally less supported (Alves-Costa et al., 2022; Connelly, Fear, Morrison, Hennelly, & Smith, 2017). Lack of enquiry by professionals was a recurrent observation in participant narratives and no participants described a process of risk assessment for IPVA, which echoes findings from research among professionals who work with military personnel (Sparrow et al., 2020) as well as civilian research (Rose et al., 2011). Significantly, most participants did not seek help, which aligns with research on help-seeking for mental health difficulties among military personnel (Greene-Shortridge et al., 2007; Hom et al., 2017; Weiss & Coll, 2011). Furthermore, certain services or interventions, such as the police (both civilian and military), IPVA charities and perpetrator programs, were not part of participants’ help-seeking journeys. Support service uptake appeared to be more common among those reporting more severe IPVA experiences. However, normalisation and minimisation of IPVA was present across participant accounts (Lane et al., 2022a, 2023) and the lack of help-seeking by the majority of participants is of concern.

The relationship between alcohol, mental health difficulties and IPVA has been shown to be complex in both military and civilian samples, with mental health difficulties found to be both a risk factor for and a consequence of IPVA (Campbell, 2002; MacManus et al., 2022; Sparrow, Kwan, Howard, Fear, & MacManus, 2017; Spencer et al., 2019). The complex interplay was illustrated in the current study by participants often turning to mental health support before help for IPVA and/or discussing help-seeking for both mental health and IPVA interchangeably, drawing links between the two. This was especially prominent for those reporting IPVA perpetration. This points to the central role mental health services hold in identifying IPVA and offering support for military personnel, and suggests a systemic as well as an individual approach is needed. It also highlights the widespread need for upskilling staff working with military communities and in welfare services as well as in civilian services, in line with research among civilian samples (Trevillion et al., 2014). Tackling IPVA and mental health difficulties in the military will likely span multiple policies and would benefit from input from those with understanding and oversight of both.

As found when exploring IPVA experiences among this cohort (Lane et al., 2022a), few gender differences in help-seeking experiences were identified, although heightened barriers relating to shame were observed for male participants reporting IPVA victimisation and perpetration. Whilst this contrasts with some civilian literature finding females more likely to seek help for IPVA victimisation (e.g., Cho, Seon, Han, Shamrova, & Kwon, 2020), our findings of frequent bidirectional IPVA support the movement aiming to dilute the rigid, often gendered, victim/perpetrator binary (Bates, 2016). Perhaps illustrative of a propensity for older individuals to avoid help-seeking (Fleming & Resick, 2017), some barriers to help-seeking appeared amplified among older participants and those who were ex-serving. However, we must acknowledge there may be slight cohort differences and the narratives of older participants and those ex-serving may not reflect recent efforts by the Ministry of Defence to raise awareness of IPVA and improve their response through their recent strategy (Ministry of Defence, 2018) and the Defence whole-force policy (Ministry of Defence, in press).

## Strengths and Limitations

The present study provides much needed qualitative exploration of UK military personnel’s experiences of help-seeking for IPVA, importantly also providing a voice to male victim-survivors, a group currently under-researched (Sparrow et al., 2018). Nonetheless, the data may be limited by social desirability bias, which could impact military personnel’s disclosure within interviews and contribute to possible minimisation or normalisation of violence previously highlighted in this cohort (Lane et al., 2022a, 2023). Interchangeable discussions of help-seeking for mental health and help-seeking for IPVA and perceptions that mental health difficulties were key drivers for IPVA in participant relationships (see Lane et al., 2022a) limited our ability to draw out data on help-seeking for IPVA specifically. Furthermore, subgroup analysis (for example by rank, serving status, and engagement status) was unfortunately limited due to the complexity of the data; for example, many participants reported different relationships with differing IPVA patterns. In addition, the present research recruited a predominantly White British sample reporting heterosexual relationships and most participants had previously deployed. The vast majority of military personnel are males, therefore it is not surprising that this study only recruited a limited number of female military personnel, though we attempted to oversample females. Further investigation is warranted to explore differences in experiences across groups and to explore the experiences of dual military couples, LGBT couples, and those from ethnic minority backgrounds in more depth.

## Recommendations and Conclusions

Given previous findings of a significant relationship between institutional response and victim-survivors of military sexual assault’s willingness to engage with services (Monteith et al., 2021), our findings support the sustained efforts by the UK Ministry of Defence to improve pathways to support for IPVA and endorse recent uplifts in staff training (Ministry of Defence, 2018). However, structural approaches have been argued to be overly simplistic in promoting health interventions and tackling culture change (Lieberman et al., 2013), pressing for a corresponding shift at the individual levels of the model to enact meaningful change. Overcoming these barriers requires a multifaceted approach to tackling barriers at each level of the social ecological model, i.e., a whole systems approach or multilevel intervention. The hierarchical, patriarchal culture within the military with emphasis on toughness filters into the home and intimate relationships and efforts to effect change at a cultural level, if they are to have lasting effect, will need to use gender transformative strategies which tackle root causes of gender inequality and unequal power relations and include relationship strengthening work (Kerr-Wilson et al., 2020). Mandatory training for all military staff would support increased understanding of IPVA, its identification, risk assessment and management. Additional courses should be completed by management and health and welfare professional groups given their position as gatekeepers to support and their role in the identification and management of IPVA. This could be complemented by interventions and creative efforts aiming to reduce stigma associated with help-seeking, as effectively piloted among UK and US military personnel (Cornish et al., 2019; Jones et al., 2014). Our findings also call for wider advertisement of support services for IPVA and a simplification of pathways to and thresholds for accessing support. Military personnel narratives highlight the importance of feeling that their experiences are understood, and their disclosures remain confidential. Establishing specialist and confidential services with established security clearances, avenues to support separate to military/chain of command and anonymous peer-support groups may offer alternative routes to support which align more with the needs of military personnel.

This study explored the experiences of and barriers to help-seeking for IPVA among UK military personnel. The findings indicate the additional barriers to help-seeking for IPVA experienced by military personnel as a result of military culture and the military environment. Participants experiences suggest that change is needed at all levels of their social ecological model, i.e., a whole systems approach, to reduce barriers relating to factors such as stigma and hypermasculine attitudes. Mandatory staff training and military-wide campaigns to raise awareness and understanding of IPVA and of support available are basic and essential components of any military IPVA strategy. Efforts to reduce barriers to accessing support for IPVA and ensure that the needs of military personnel are met will soon no longer be optional once they are enshrined in the new Government Ministry of Defence Domestic Abuse policy.
